# Coupling growth kinetics modeling with machine learning reveals microbial immigration impacts and identifies key environmental parameters in a biological wastewater treatment process

**DOI:** 10.1186/s40168-019-0682-x

**Published:** 2019-04-17

**Authors:** Ran Mei, Jinha Kim, Fernanda P. Wilson, Benjamin T. W. Bocher, Wen-Tso Liu

**Affiliations:** 1 Department of Civil and Environmental Engineering, University of Illinois at Urbana-Champaign, 3207 Newmark Civil Engineering Laboratory, 205 North Mathews Ave, Urbana, IL 61801 USA; 2 Petrochemicals Technology, BP America, Naperville, IL 60563 USA

**Keywords:** Immigration impact, Active population, Machine learning

## Abstract

**Background:**

Ubiquitous in natural and engineered ecosystems, microbial immigration is one of the mechanisms shaping community assemblage. However, quantifying immigration impact remains challenging especially at individual population level. The activities of immigrants in the receiving community are often inadequately considered, leading to potential bias in identifying the relationship between community composition and environmental parameters.

**Results:**

This study quantified microbial immigration from an upstream full-scale anaerobic reactor to downstream activated sludge reactors. A mass balance was applied to 16S rRNA gene amplicon sequencing data to calculate the net growth rates of individual populations in the activated sludge reactors. Among the 1178 observed operational taxonomic units (OTUs), 582 had a positive growth rate, including all the populations with abundance > 0.1%. These active populations collectively accounted for 99% of the total sequences in activated sludge. The remaining 596 OTUs with a growth rate ≤ 0 were classified as inactive populations. All the abundant populations in the upstream anaerobic reactor were inactive in the activated sludge process, indicating a negligible immigration impact. We used a supervised learning regressor to predict environmental parameters based on community composition and compared the prediction accuracy based on either the entire community or the active populations. Temperature was the most predictable parameter, and the prediction accuracy was improved when only active populations were used to train the regressor.

**Conclusions:**

Calculating growth rate of individual microbial populations in the downstream system provides an effective approach to determine microbial activity and quantify immigration impact. For the studied biological process, a marginal immigration impact was observed, likely due to the significant differences in the growth environments between the upstream and downstream processes. Excluding inactive populations as a result of immigration further enhanced the prediction of key environmental parameters affecting process performance.

**Electronic supplementary material:**

The online version of this article (10.1186/s40168-019-0682-x) contains supplementary material, which is available to authorized users.

## Background

Microbial immigration is a widespread process in natural and engineered environments and plays important roles in shaping the downstream microbial community. In natural lake environments, bacterioplankton assemblage compositions were influenced by bacteria in the inlet running water [1], treated or untreated sewage discharge [2], and even atmospheric deposition [3]. In rivers, planktonic and sedimentary microbial communities can be influenced by the immigration between each other [4]. Microbial immigration in engineered systems has also been reported in tap water bacterial community where the composition after stagnation was strongly affected by microbes in city water and pipe biofilms [5]. In wastewater treatment processes, feed sludge affected lab-scale anaerobic digester community structure and functional stability by sustainably introducing specific taxa [6]. In a lab-scale three-stage moving bed biofilm reactor, an increase in species richness was observed in the last stage as the result of immigration from the influent and first two stages [7]. In full-scale systems, significant immigration of ammonia-oxidizing bacteria from the upstream nitrifying trickling filter to the downstream activated sludge reactor was observed using NO_2_^−^ as a measurement of transportation [8]. In full-scale anaerobic digesters, feed microbial biomass was observed to account for a significant portion of the digester communities [9, 10]. Albeit weak, immigration impact was observed from the influent wastewater to the downstream activated sludge based on community diversity and composition [11].

While microbial immigration is widely observed, it is difficult to quantify its impact on the downstream microbial community assembly with currently available approaches. The neutral community model fits the observation frequency of different taxa as a function of mean relative abundance and calculates an immigration probability *m* of the entire community [12]. Although been applied in various environments [4, 5, 13], the model does not provide population-level resolution on how a specific downstream community member is affected by immigration. Another method, microbial source tracking, estimates the proportion of taxa in the downstream community that come from multiple upstream environments [14–16]. However, this method assumes that all the observed microbial populations in the downstream community come from upstream environments and ignores the fact that some active microorganisms will undergo rapid reproduction independent from immigration. Other studies simply count shared species between upstream and downstream communities, which is usually visualized with a Venn diagram [11, 17]. This approach could only provide a numerical summary of potential immigrants and ignores their different fates in the downstream environment. While acting as a continuous seed to inoculate active players to the downstream environment [8], the upstream immigrants can also introduce a significant amount of seemingly abundant but inactive organisms [9]. These challenges call for a better evaluation of the in situ activities of individual immigrants in the downstream environment during quantifying microbial immigration impacts.

Differentiating immigrant populations that actively or inactively occupy the downstream system can better predict the relationship between the microbial community and environmental parameters, which is the key mission in many ecological studies. Various methods have been applied to identify the key environmental parameters. Clustering methods such as unweighted pair group method with arithmetic mean and k-means clustering were used to group communities based on population abundance and correlate different clusters with distinct parameters in anaerobic digester [10] or human intestinal microbiome [18]. Ordination methods such as principal components analysis, non-metric multidimensional scaling, correspondence analysis, and redundancy analysis treat population abundance as multiple variables and characterize similar communities based on shared parameters in anaerobic enrichment cultures [19], fish gut [20], human throat [21], and bioreactors [22]. Compared to clustering and ordination methods, supervised learning methods (including classification and regression that can predict unlabeled samples based on labeled microbial communities) are less widely used. Overall, all those methods rely on the assumption that abundant populations are active and contributing to system function. Such community-environment correlation can be biased if the system contains inactive populations that immigrate from the upstream, which can be reduced by assessing immigration impact with consideration of microbial activity.

In this study, we attempted to address two key questions: “can we quantitatively assess the activities of immigrants after entering the downstream ecosystem?” and “can we better predict key environmental parameters from microbial community composition?” To answer these questions, we analyzed a full-scale system that couples an upstream upflow anaerobic sludge blanket (UASB) reactor and downstream activated sludge reactors to treat wastewater containing purified terephthalic acid (PTA), an important petrochemical product. We used a mass balance model with amplicon sequencing [23] to calculate the in situ activity of each community member and quantify the intensity of immigration from the upstream anaerobic reactor to the downstream activated sludge. A supervised learning regressor [24] was used to predict important environmental parameters after considering immigration impact.

## Methods

### Sampling the activated sludge system

Sludge samples were taken from a full-scale PTA-wastewater treatment facility that operated an upstream UASB reactor and three downstream parallel activated sludge processes (including aeration tanks and clarifiers) (Additional file 1: Figure S1). Six sampling events were conducted during the spring and summer of 2017. To profile the microbial community as a continuum, 11 samples were taken along the process at each sampling event (Additional file 1: Figure S1): one sample from the middle of the UASB reactor sludge bed, one sample from the UASB effluent before being split and fed into the three aeration tanks, two samples from each of the three parallel aeration tanks (at different locations as replicates), and one sample from each of the three clarifier underflows. After collection, the samples were shipped to the laboratory at the University of Illinois at Urbana-Champaign (UIUC) on ice overnight and stored at − 80 °C prior to further analyses. Operation or physicochemical parameters, including temperature, dissolved oxygen (DO), ammonia, phosphate, and total organic carbon (TOC), were monitored and provided by the facility managers (detailed methods are available in Additional file 1). Phosphate measurement at one third of the sampling locations was not available. Terephthalic acid concentration of the samples was measured in UIUC lab using a HPLC with Agilent ZORBAX eclipse XDB-C18 column [25]. Total suspended solids (TSS) and volatile suspended solids (VSS) of the samples were measured according to the standard protocol [26].

### DNA extraction, PCR, and 16S rRNA gene sequencing

16S rRNA gene amplicon sequencing was performed as described previously [27]. Briefly, DNA was extracted using the FastDNA SPIN Kit for Soil, and 16S rRNA gene was amplified with the Bacteria/Archaea universal primer sets Univ515F/Univ909R that target V4-V5 region [28]. Purified and pooled PCR amplicons were sequenced on an Illumina Miseq platform using the v3 chemistry at the Roy J. Carver Biotechnology Center at UIUC.

### Sequence analysis and supervised learning regression

16S rRNA gene sequences were analyzed with the QIIME 2 platform (v2018.6) [29]. Raw sequences were first processed using DADA2 [30], including quality filtering, denoising, paired-end sequence merging, and chimera filtering. DADA2 generated unique amplicon sequence variants that were equivalent to 100% similarity operational taxonomic units (OTU) in the conventional practice. In this publication, we still use the term OTU for the purpose of simplicity. Taxonomy was assigned using q2-feature-classifier [31] customized for the primer set used in this study with Silva SSU database release 132 [32]. Multiple sequence alignment and phylogenetic tree construction were performed using the QIIME 2 plugin q2-phylogeny. For downstream diversity analysis, the OTU table was rarefied to 18,578 sequences per sample determined by the sample with least sequences. Alpha and beta diversity analyses were performed using the QIIME 2 plugin q2-diversity. Supervised regression of operation parameters on community compositions was performed using the QIIME2 plugin q2-sample-classifier with default settings [24]. Eighty percent of the samples were randomly picked to train the regressor. The remaining 20% of the samples were used to validate classification accuracy of the optimized regressor. The neutral model that fit OTU frequency to mean relative abundance [12] was applied to calculate immigration probability using the nlsLM function in R package minpack.lm. During calculation, OTU frequency was counted among all the activated sludge samples and mean relative abundance was averaged. Redundancy analysis was performed using R package vegan, and the significance of constraints was tested with permutation tests (999 permutations) [33].

### Reactor kinetics

The mass balance calculation was performed as described previously [34]. The control volume was defined as one aeration tank and its downstream classifier. The change of mass (cell number) of a given microbial population *x* in this control volume was contributed by incoming biomass from the UASB effluent, outgoing biomass in the wasted sludge and clarifier effluent, and net growth in the aeration tank. The mass balance can be described as:

$$ \frac{d{N}_{x,\mathrm{AS}}}{dt}={n}_{x,\mathrm{UASB}}-{n}_{x,\mathrm{waste}}-{n}_{x,\mathrm{eff}}+{\mu}_x{N}_{x,\mathrm{AS}} $$

where *N*_*x*, AS_ is the cell number of population *x* in the aeration tank; *n*_*x*, UASB_ is the number of *x* in the UASB effluent entering aeration tank per day [d^−1^]; *n*_*x*, waste_ is the number of *x* in the wasted sludge leaving the system per day [d^−1^]; *n*_*x*, eff_ is the number of *x* in the clarifier leaving the system per day [d^−1^]; and *μ*_*x*_ is net growth rate constant of *x* [d^−1^]. The number of *x* is obtained by multiplying the total cell number in the sample, which was approximated by concentration of volatile suspended solids [34], and the relative abundance of *x* in the microbial community, which was calculated based on 16S rRNA gene sequences. Detailed steps for calculation are available in Additional file 1. Growth rates of individual populations were calculated for the three activated sludge systems on different dates separately and averaged for further analyses.

## Results

### Mass balance identified active populations and indicated marginal immigration impact

In total, 1178 OTUs were observed and rarefaction curves indicated that sufficient sequencing depth was achieved (Additional file 1: Figure S2). To calculate the growth rate of individual populations, three assumptions were made and validated based on system performance data. First, the system was assumed to be operated at steady state at the time of sampling, which was supported by the stable TOC concentrations in the aeration tanks (Additional file 1: Figure S3a–c). The first sampling event in tank A was excluded for the analysis due to unstable TOC concentrations during the sampling period (Additional file 1: Figure S3a). Second, the biological activity in the clarifiers was negligible, and all the growth was assumed to occur in the aeration tank, which was supported by the similar TOC concentrations in the aeration tank and clarifier (Additional file 1: Figure S3a–c). Third, the biomass in the clarifier effluent was negligible, which was supported by the significantly low TSS concentrations in the clarifier effluent compared to the TSS of wasted sludge (Additional file 1: Figure S3d).

**Fig. 1:**
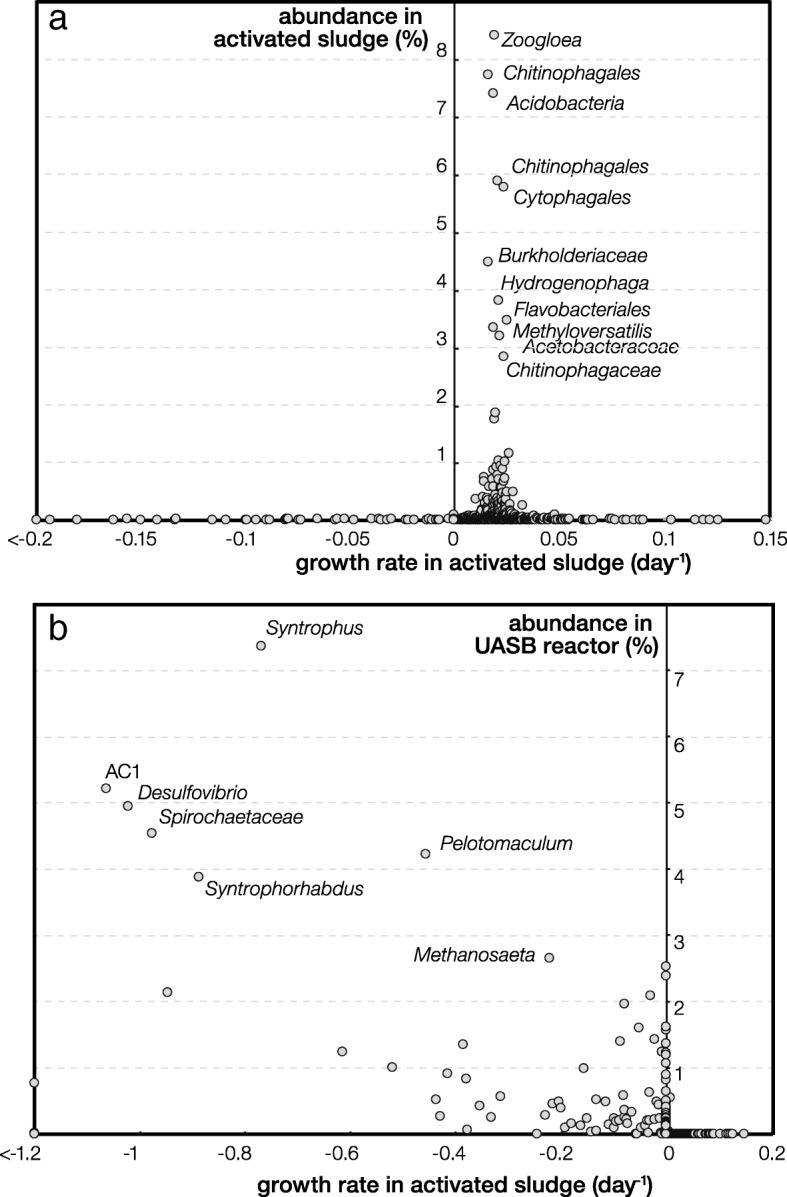
Growth rate of populations observed in the activated sludge reactors. The *y*-axis in **a** denotes abundance in aeration tank, and in **b** abundance in UASB reactor

The growth rate calculation results are presented in Fig. 1. In total, 586 OTUs had a positive growth rate in activated sludge and accounted for 99% of the total sequences. OTUs with abundance higher than 0.1% all had a positive growth rate (Fig. 1a). This active subset of the community (i.e., active community) included the most abundant populations that were commonly detected in activated sludge taxa such as *Zoogloea*, *Chitinophagales*, *Flavobacteriales*, and *Burkholderiaceae*. The remaining 592 OTUs had a growth rate ≤ 0. This inactive subset of the community included populations that were abundant in the upstream UASB reactor, such as *Syntrophus*, *Syntrophorhabdus*, *Pelotomaculum*, and *Methanosaeta*, which were obligate anaerobes (Fig. 1b). They exhibited a significant decrease in abundance in aeration tanks and collectively contributed to 1% of the total sequences in activated sludge. Such marginal immigration impact by the upstream UASB community was supported by the small immigration probability *m* (0.028) for the entire activated sludge community calculated by fitting OTU frequency as a function of mean relative abundance using the neutral community model (Additional file 1: Figure S4). This suggested that local reproduction was the dominant process during community assemblage with a high probability at 0.972. A Venn diagram also revealed a similar result that only 78 OTUs were shared between UASB and activated sludge community and accounted for 0.4% of total sequences in obtained activated sludge (Additional file 1: Figure S5).

### Active populations better predicted environmental parameters

We applied a supervised learning regressor to predict environmental parameters based on microbial community composition (i.e., OTU abundance). A selected fraction of the samples was used to train the regressor, and the remaining samples were used to assess the prediction accuracy by comparing observed and predicted values. Intuitively, we used samples from two of the three parallel aeration tanks for training and used the third tank for testing. However, poor prediction accuracy was observed, suggested by the large discrepancies between observed and predicted values (Additional file 1: Figure S6). None of the squared linear least-squares regression coefficients (*R*) exceeded 0.7, except for temperature.

**Fig. 2:**
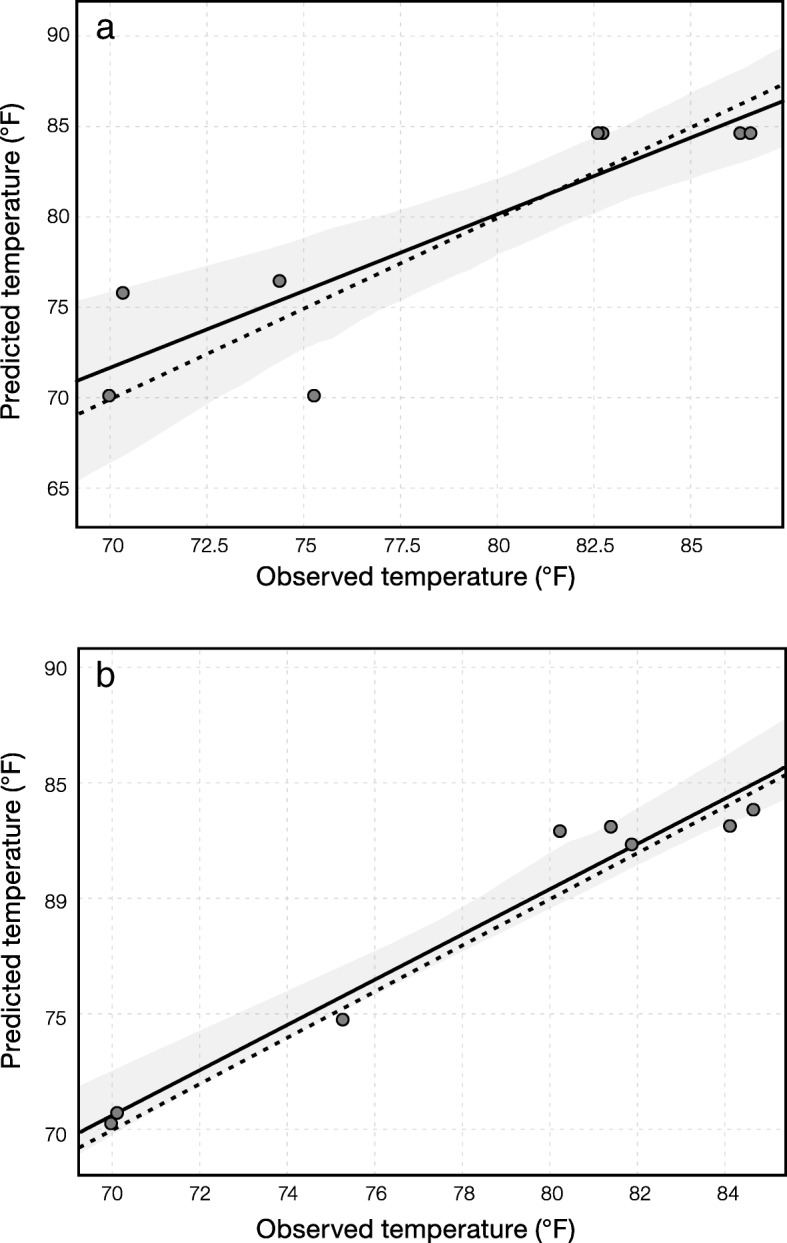
Prediction of temperature based on **a** the entire community and **b** the active community. The *x*- and *y*-axis denote observed and predicted temperature, respectively. The dashed line represents 1:1 ratio of observed and predicted value. The solid line represents the fitted trend of the observed and predicted value. The gray shadow represents 95% confidence interval with 100 times of estimation

Instead of using two thirds of the samples for training the regressor, we pooled all the samples from the three tanks and tested the optimal fraction of samples for training the regressor. Based on the prediction accuracy, including mean squared error, *R* squared, and slope (Additional file 1: Table S1), it was shown that using 80% of the samples for regressor training and 20% samples for accuracy testing produced the optimal results. When the entire microbial community was used to train the regressor, temperature had higher predictability than other parameters. The fitted line of the predicted value versus observed value had a *R* squared of 0.780 and a slope of 0.848 (Fig. 2a). The prediction of phosphate also had high *R* squared (0.910) and slope (0.761), but the 95% confidence interval was comparably large due to missing one third of the phosphate concentration data (Additional file 1: Figure S7). Other parameters, i.e., TOC, DO, ammonia, and TA concentration, had relatively poor predictability with *R* squared and slope lower than 0.7.

We further used the active community (i.e., OTUs with positive growth rates) in the activated sludge reactor to train and test the regressor (Additional file 1: Figure S8). Temperature was still the most predictable parameter with almost perfect accuracy. The *R* squared of 0.955 and slope of 0.980 were very close to the 1:1 observed/predicted line and were both higher than the values when the entire community was used (Fig. 2b). An improved confidence interval was also observed throughout the entire prediction zone. For other parameters, the prediction also improved as suggested by lower mean squared error, higher *R* squared, and higher slope in most cases (Table 1).

**Table 1:** Comparison of prediction accuracy between the entire community and active community. MSE stands for mean square error

Parameter	Entire community				Active community				Active/Entire		
	MSE	*R* squared	*p* value	Slope	MSE	*R* squared	*p* value	Slope	MSE	*R* squared	Slope
Temp.	9.375	0.780	0.004	0.848	3.608	0.955	0.000	0.980	0.385	1.225	1.155
TOC	7.203	0.671	0.013	0.425	2.606	0.843	0.001	0.615	0.362	1.255	1.445
DO	1.037	0.378	0.105	0.187	0.464	0.195	0.274	0.537	0.447	0.515	2.877
NH_4_^+^	0.006	0.659	0.014	0.587	0.006	0.541	0.038	0.726	0.997	0.820	1.238
PO_4_^3−^	0.116	0.910	0.012	0.761	0.059	0.946	0.005	0.720	0.505	1.041	0.946
TA	0.000	0.120	0.400	0.125	0.000	0.531	0.040	0.153	1.750	4.416	1.219

### Redundancy analysis identified active populations associated with environmental parameters

**Fig. 3:**
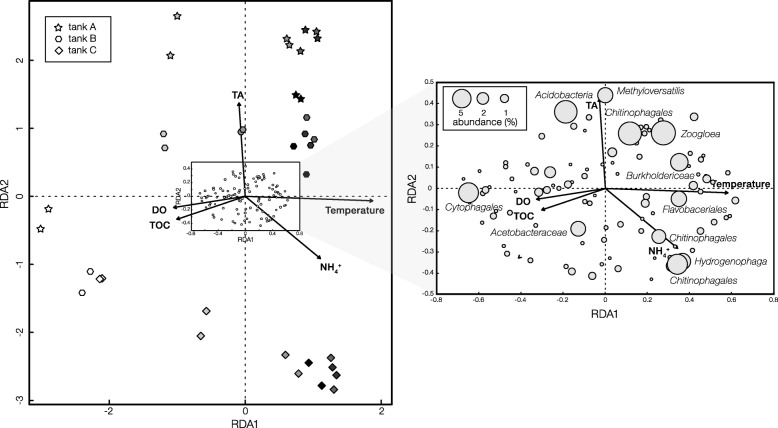
Redundancy analysis based on the abundance of active populations in activated sludge. The left panel is a tri-plot of samples, OTUs, and performance parameters. Stars, hexagons, and diamonds represent samples from the three parallel tanks. Gray scale of the fill color represents time scale of the sampling from February to June. Small dots in the middle represent OTUs, which are zoomed in on the right panel. Size of the circle represents relative abundance in activated sludge. The length of an environmental parameter arrow in the resulting ordination indicates the strength of the relationship of that parameter to community composition

Redundancy analysis was performed on the active community to identify which populations were associated with individual environmental parameters (Fig. 3). Permutation tests indicated statistical significance (*p* < 0.05) of the associations for temperature, NH_4_^+^, TOC, and TA, but not for DO (*p* = 0.259). In terms of community structure, all the three parallel aeration tanks followed similar evolution trajectory along sampling time from spring to summer (light to dark symbols on the left panel). This trend was consistent with the direction of increasing temperature and agreed with the aforementioned result that temperature was the most predictable environmental parameters to describe the community structure. The impact of temperature was further observed on individual OTUs, where the most dominant OTUs were distributed in the direction of increasing temperature (right panel). The only dominant OTU that was located in the opposite direction of temperature was related to *Cytophagales*, which might prefer lower temperature. The direction of increasing temperature opposed that of TOC, implying higher temperature was associated with higher consumptions of organic carbon. TA concentration was independent from the temperature effect, and abundant OTUs related to *Methyloversatilis* and *Acidobacteria* were associated with higher TA concentration.

## Discussion

Microbial immigration constantly contributes to community assemblage in natural and engineered microbial ecosystems. However, the intensity of its impact varies. Abundant populations in the upstream process can play active or inactive roles in the downstream. This could not be effectively addressed by many previous studies [4, 5, 11, 13–17]. Coupling a mass balance model with amplicon sequencing data allows the calculation of the net growth rate of individual community members, enabling the differentiation between actively growing populations and non-growing ones. This further allows to evaluate the impact of microbial immigration on the downstream system by addressing whether the upstream process can introduce microbial populations that will remain active in the downstream system. This strategy was first used to reveal that influent wastewater introduced populations that could actively grow in the receiving activated sludge system as well as non-growing populations [23]. A more significant immigration impact was observed in anaerobic digesters where 25% of the sequences were associated with non-growing populations introduced by the feed microbial biomass [34]. In contrast to the previous two cases of immigration from raw wastewater (aerobic) to activated sludge (aerobic) environment and from activated sludge (aerobic) to anaerobic digester (anaerobic) environment, the present study examined another distinct immigration case from UASB reactor (anaerobic) to activated sludge (aerobic) environment. Non-growing populations only occupied 1% of the activated sludge community, and abundant populations in the upstream anaerobic reactor did not grow in activated sludge due to a significant change in environmental condition (anaerobic vs. aerobic). The marginal immigration impact observed could also be reflected by the small migration probability in the Sloan model and small number of shared OTUs in Venn diagram (Additional file 1: Figure S4 and Additional file 1: Figure S5).

The active populations in activated sludge processes, which are not influenced by upstream immigration, can be viewed as the key players of important ecological functions. Methanol was one of the major components in PTA wastewater [35], and an active population related to *Methyloversatilis* that could utilize methanol as carbon and energy source [36] was observed. Populations associated with *Cytophaga*, *Flavobacterium*, and *Chitinophaga* have been reported to degrade soluble microbial products in anaerobic reactor effluent [37]. OTUs related to these taxa that might scavenge anaerobic by-products that were present activated sludge given that TA and BA accounted for only 31–69% of all TOC (Additional file 1: Table S2).

The inactive populations, although not abundant, were also detected and accounted for 50.2% of the 1178 OTUs observed in activated sludge. This suggested that functionally active populations only accounted for half of the observed species richness, which could not be revealed by using approaches currently available. Analyzing 16S rRNA gene sequences alone could only provide evidences of detectable microorganisms but not necessarily active ones and could be influenced by the rRNA copy numbers associated with different microorganisms [38]. Meta-omics approaches (e.g., metagenomic and metatranscriptomic) could shed lights on metabolic function at multiple levels, but the information obtained relies on the availability of reference genomes with high-quality annotations, which are still scarce [39]. Stable-isotope probing coupled with molecular biomarker sequencing can associate microbial identity with function [40], but is limited by its throughput to monitor all populations. Also, cross-feeding phenomena can sometimes restrict its application. Fluorescence in situ hybridization can provide evidence of presence and activity, especially by coupling with microautoradiography to visualize uptake of specific substrates [41]. However, applications of such target-specific methods are limited to a few pre-determined microorganisms. Our findings showed that the mass balance model coupled with amplicon sequencing provides a novel and high-throughput approach to effectively characterize microbial activity compared to the aforementioned methods, and can be widely applied in environmental and applied microbiology.

Our study also highlighted that the application of a supervised learning regressor could identify predictive and important environmental parameters. When the regressor was trained on the active subset of the community instead of the entire community, the prediction accuracy of environmental parameters was greatly improved. This is reasonable because active populations are primarily responsible for important ecological functions. Inactive populations likely did not contribute to key ecological functions and thus could be regarded as baseline noise of the community. If the immigration impact is strong, for example, if a large fraction of the community is occupied by non-growing populations introduced from the upstream process, such an approach can be even more effective. Last, we demonstrated that sufficient sample numbers and comprehensive environmental parameters monitoring were critical to ensure good performance of regression, which emphasizes the necessity to perform holistic sampling and monitoring for engineered processes in future studies.

## Additional file

Additional file 1: Supplementary figures and tables. This file contains supplementary **Figures S1–S8.** and **Tables S1–S2.** (PDF 4054 kb)
