# Supplementary material for: Coupling growth kinetics modeling with machine learning reveals microbial immigration impacts and identifies key environmental parameters in a biological wastewater treatment process
Source: Microbiome. 2019 Apr 17;7:65. doi: 10.1186/s40168-019-0682-x (PMC6471889; doi:10.1186/s40168-019-0682-x)
Supplement: Supplementary file 1 — Supplementary figures and tables. This file contains supplementary Figures S1–S8. and Tables S1–S2. (PDF 4054 kb) [file 40168_2019_682_MOESM1_ESM.pdf]

Coupling growth kinetics modeling with machine learning reveals microbial immigration impacts and identifies key environmental parameters in a biological wastewater treatment process

Ran Mei, Jinha Kim, Fernanda P. Wilson, Benjamin T.W. Bocher, Wen-Tso Liu

Supplementary Materials

Supplementary methods

Calculation of net growth rate

With the mass balance model

$$\frac{dN_{x,AS}}{dt} = n_{x,UASB} - n_{x,waste} - n_{x,eff} + \mu_x N_{x,AS} \qquad \text{(S1)}$$

where

$N_{x,AS}$	cell number of microorganism x in aeration tank [-]
t	time [d]
$n_{x,UASB}$	number of x in UASB effluent entering the system per day [d^{-1}]
$n_{x,waste}$	number of x in wasted sludge leaving the system per day [d^{-1}]
$n_{x,eff}$	number of x in clarifier effluent leaving the system per day [d^{-1}]
μ_x	net growth rate of microorganism x [d^{-1}]

Several assumptions are applied:

A1. The system is running at steady state, there is no net change of cell number of x. Thus $\frac{dN_{x,AS}}{dt} = 0$.

A2. The clarifier is running efficiently and the biomass in the effluent is negligible. Thus $n_{x,eff} = 0$.

A3. The biological activity in the clarifier is negligible thus all the growth occurs in aeration tank.

With the aforementioned assumptions, equation S1 can be rearranged as

$$\mu_x = \frac{n_{x,waste} - n_{x,UASB}}{N_{x,AS}} \qquad \text{(S2)}$$

Cell number of x in wasted sludge, UASB effluent and AS can be calculated by multiplying total cell number of the sludge with the relative abundance of x that is obtained from OTU table. S2 can be expanded as

$$\mu_x = \frac{p_{x,waste} n_{waste} - p_{x,UASB} n_{UASB}}{p_{x,AS} N_{AS}} \qquad \text{(S3)}$$

n_{waste} is the total number of cells leaving the system per day with wasted sludge, and can be calculated by multiplying the daily volume of wasted sludge (Q_{waste}) and cell concentration in the wasted sludge C_{waste}. Similarly, n_{UASB} is the total number of cell entering the system per day with UASB effluent, and can be calculated by multiplying

the daily volume of UASB effluent (Q_{UASB}) and cell concentration in the UASB effluent C_{UASB}. N_{AS} is the total number of cell in the aeration tank, and can be calculated by multiplying the volume of aeration tank (V_{AS}) and cell concentration in the aeration tank C_{AS}. With these, S3 can be expanded as

$$\mu_x = \frac{p_{x,waste} Q_{waste} C_{waste} - p_{x,UASB} Q_{UASB} C_{UASB}}{p_{x,AS} V_{AS} C_{AS}} \quad \text{(S4)}$$

Here we use concentration of volatile suspended solids (VSS) of the sludge as a proximity to cell concentration. Measuring the volatile suspended solids (VSS) is a common approach to determine biomass concentration in sludge samples [1, 2]. In our previous study [3], we applied VSS concentration to estimate cell number for mass balance, which was further verified by using qPCR. The growth rate based on VSS and qPCR was statistically consistent. Therefore, we decided to keep using VSS to correlate to absolute cell number in this study. S4 can be expressed as

$$\mu_x = \frac{p_{x,waste} Q_{waste} VSS_{waste} - p_{x,UASB} Q_{UASB} VSS_{UASB}}{p_{x,AS} V_{AS} VSS_{AS}} \quad \text{(S5)}$$

The final growth rate of microorganism x is averaged using values calculated from three aeration tanks.

System performance monitored by the facility managers

Performance of the full-scale anaerobic-aerobic system was monitored by the facility managers. Phosphate concentration was measured using a SPECTRO ARCOS ICP-OES analyzer. TOC was measured using a Shimadzu TOC-L TOC analyzer. Ammonia concentration was measured according to the standard method SM4500-NH3-D2011 using a Orion 4 Star Meter/Thermo Orion High Performance Ammonia Electrode.

Reference:

1. Contreras, E.M., et al., *A modified method to determine biomass concentration as COD in pure cultures and in activated sludge systems.* Water Sa, 2002. **28**(4): p. 463-468.
2. Yuan, Q., R. Sparling, and J. Oleszkiewicz, *Waste activated sludge fermentation: effect of solids retention time and biomass concentration.* Water research, 2009. **43**(20): p. 5180-5186.
3. Mei, R., et al., *Evaluating digestion efficiency in full-scale anaerobic digesters by identifying active microbial populations through the lens of microbial activity.* Scientific Reports, 2016. **6**: p. 34090.

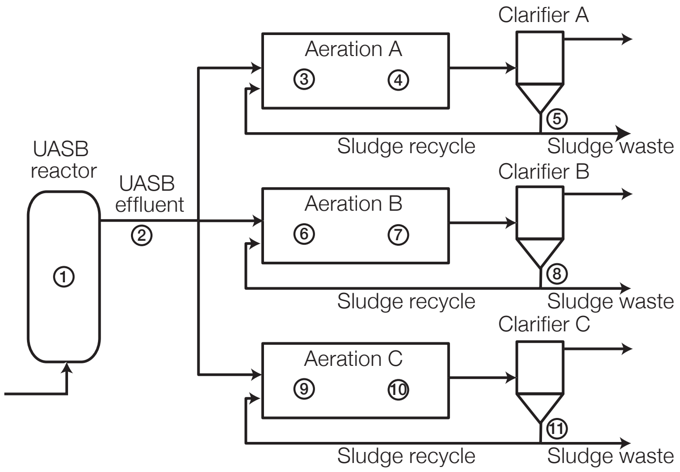

Fig. S1. System configuration and sampling locations. 1. granules from middle of UASB reactor sludge bed; 2. UASB effluent before being fed to activated sludge; 3. first stage of aeration tank A; 4. second stage of aeration tank A; 5. underflow of clarifier A; 6. first stage of aeration tank B; 7. second stage of aeration tank B; 8. underflow of clarifier B; 9. first stage of aeration tank C; 10. second stage of aeration tank C; 11. underflow of clarifier C.

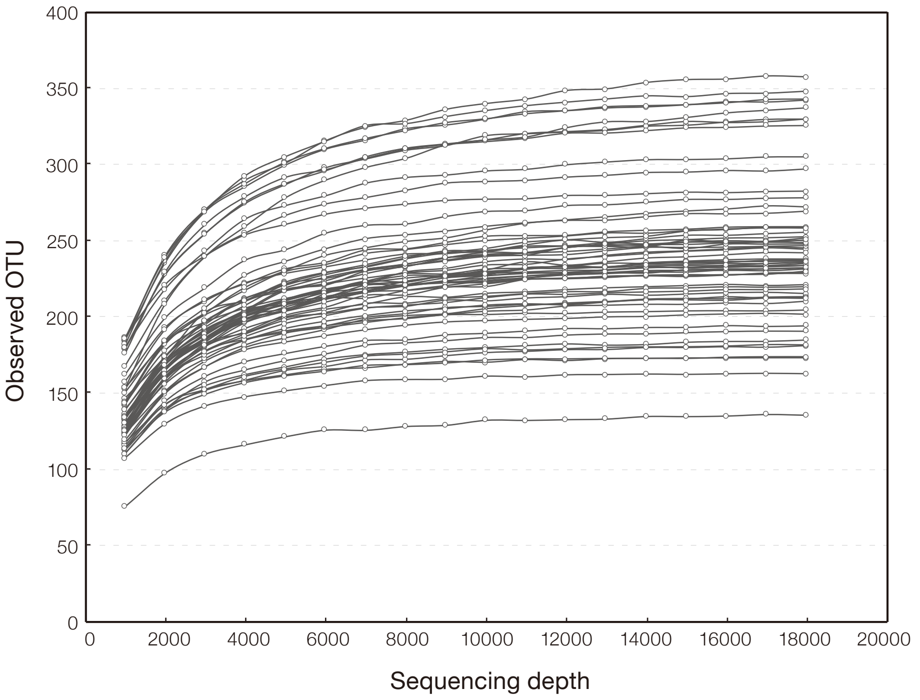

Fig. S2. Rarefaction curves of each samples based on observed OTU.

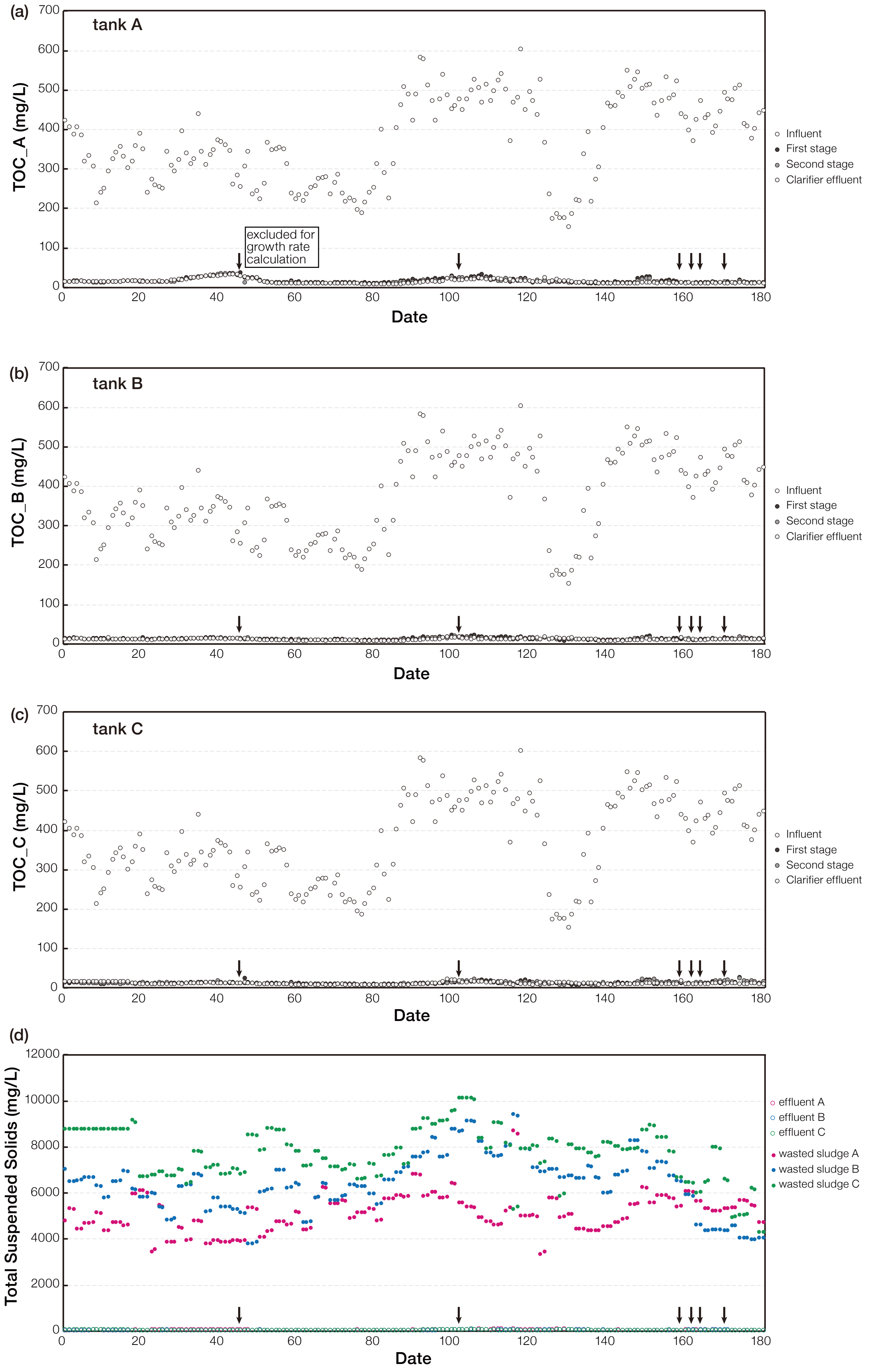

Fig. S3. System operation. (a)-(c) TOC concentrations of aeration tank A, B, and C. (d) TSS of wasted sludge and effluent of clarifiers. Arrows denote the six sampling events.

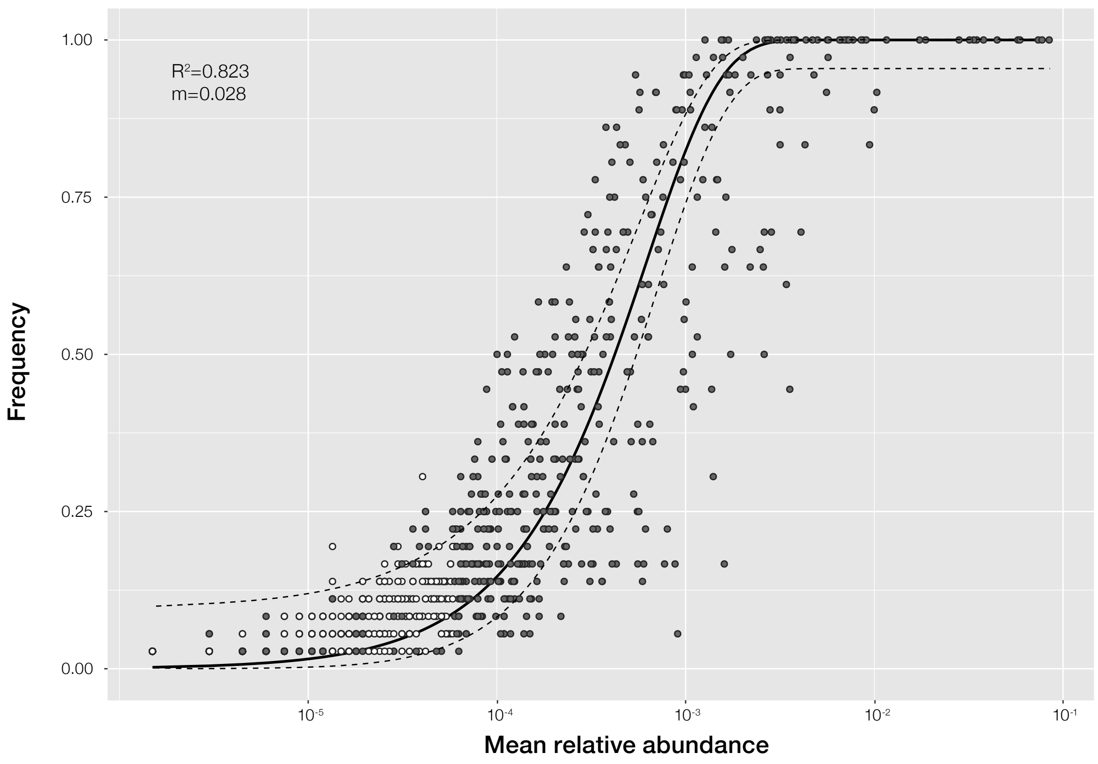

Fig. S4. Fit OTU frequency as a function of mean relative abundance using Sloan's neutral model. Dashed lines represent 95% confidence intervals around the model prediction. Solid circles represent active populations. Open circles represent inactive populations.

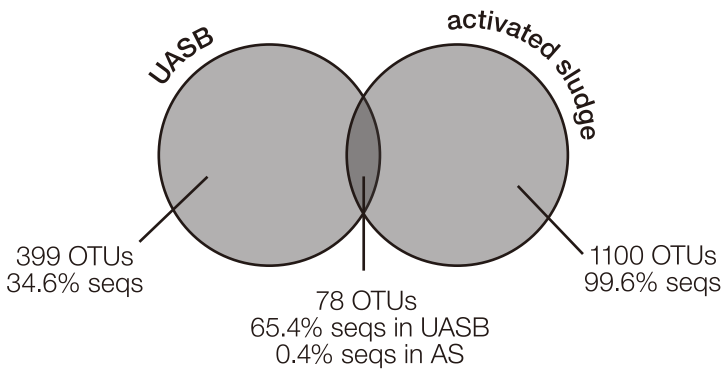

Fig. S5. Venn diagram shows shared OTU between UASB reactor and activated sludge.

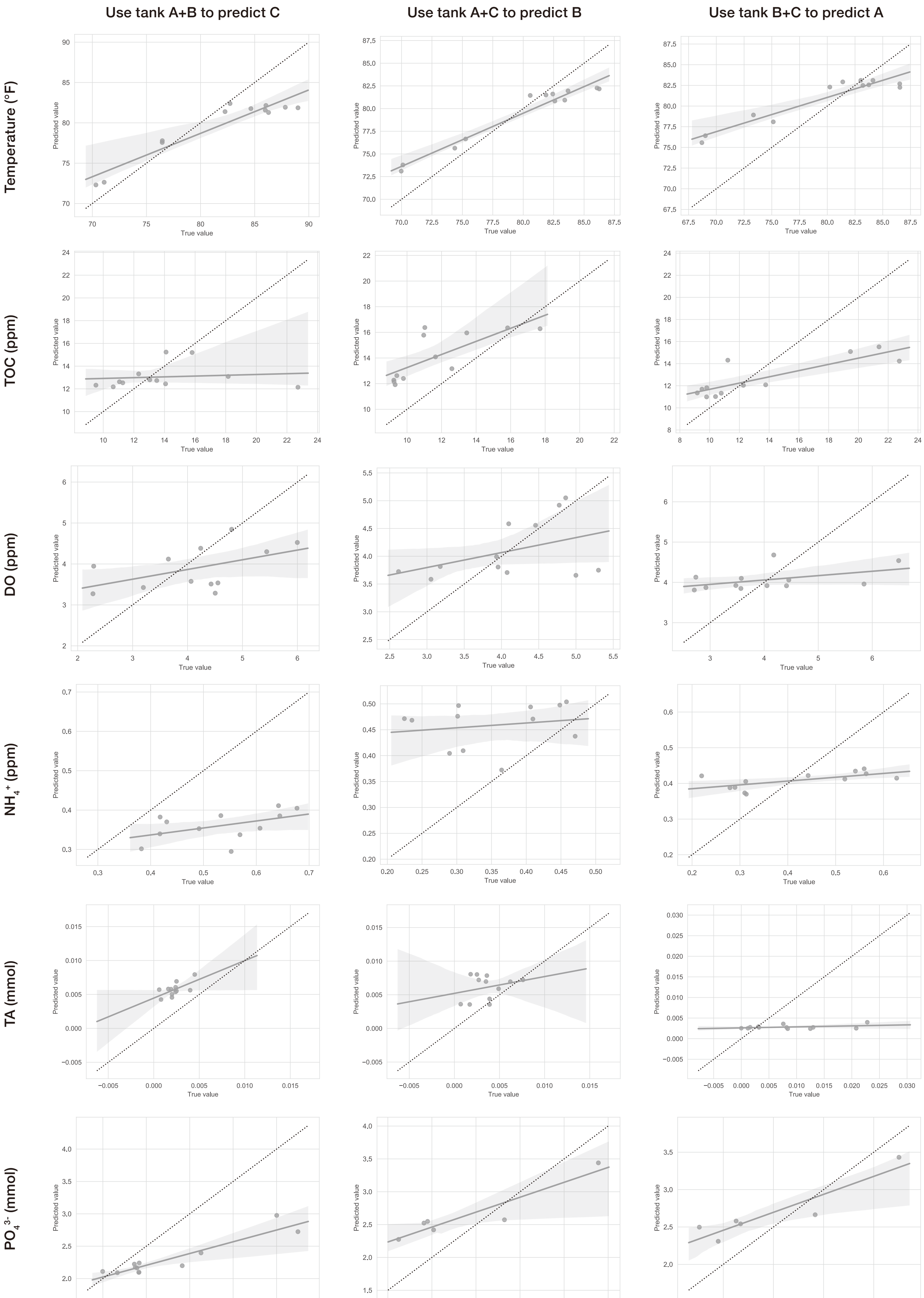

Fig. S6. Using two aeration tanks to predict the operation parameters of the third tank

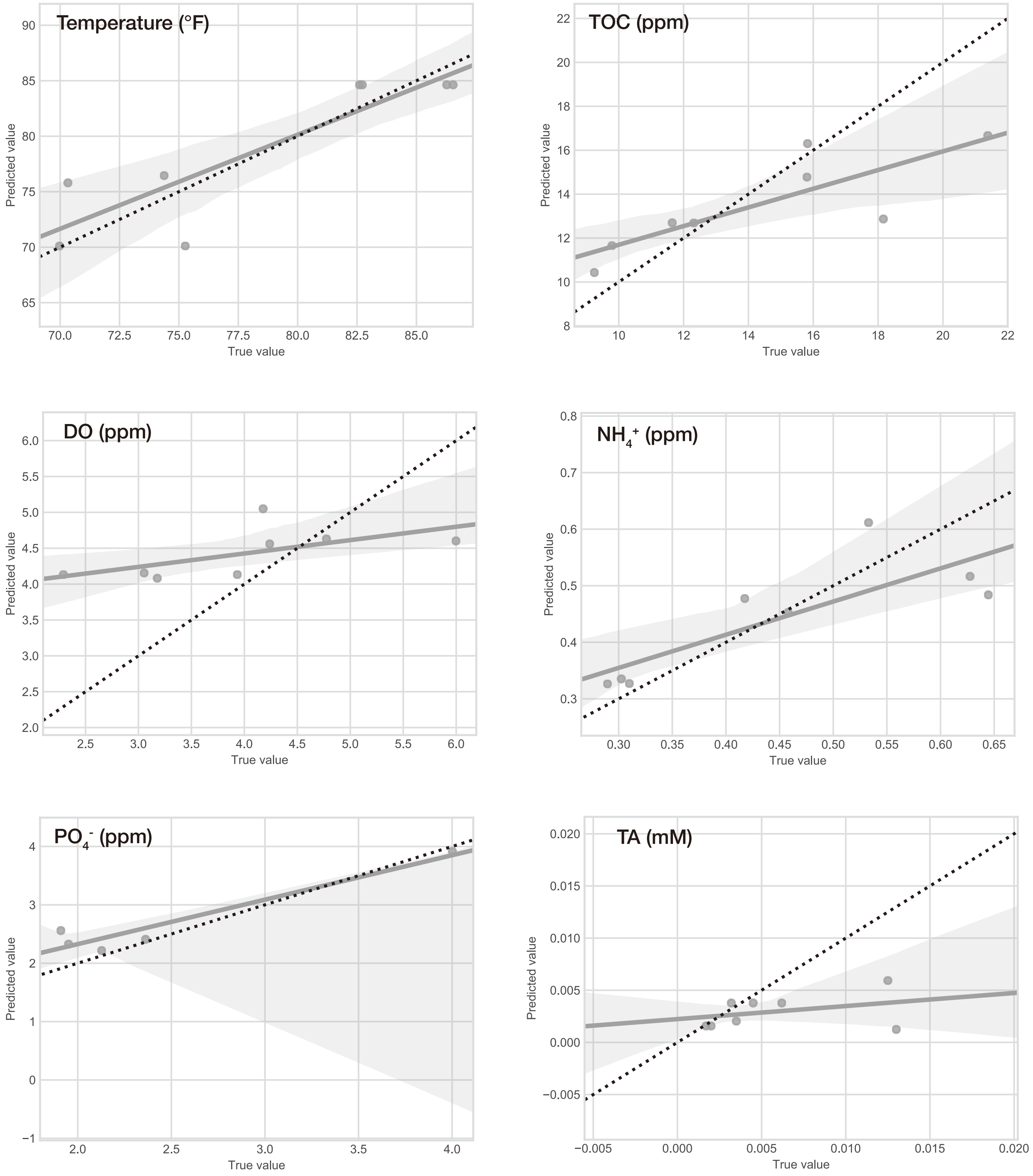

Fig. S7. Prediction of operation parameters based on entire community compositions.

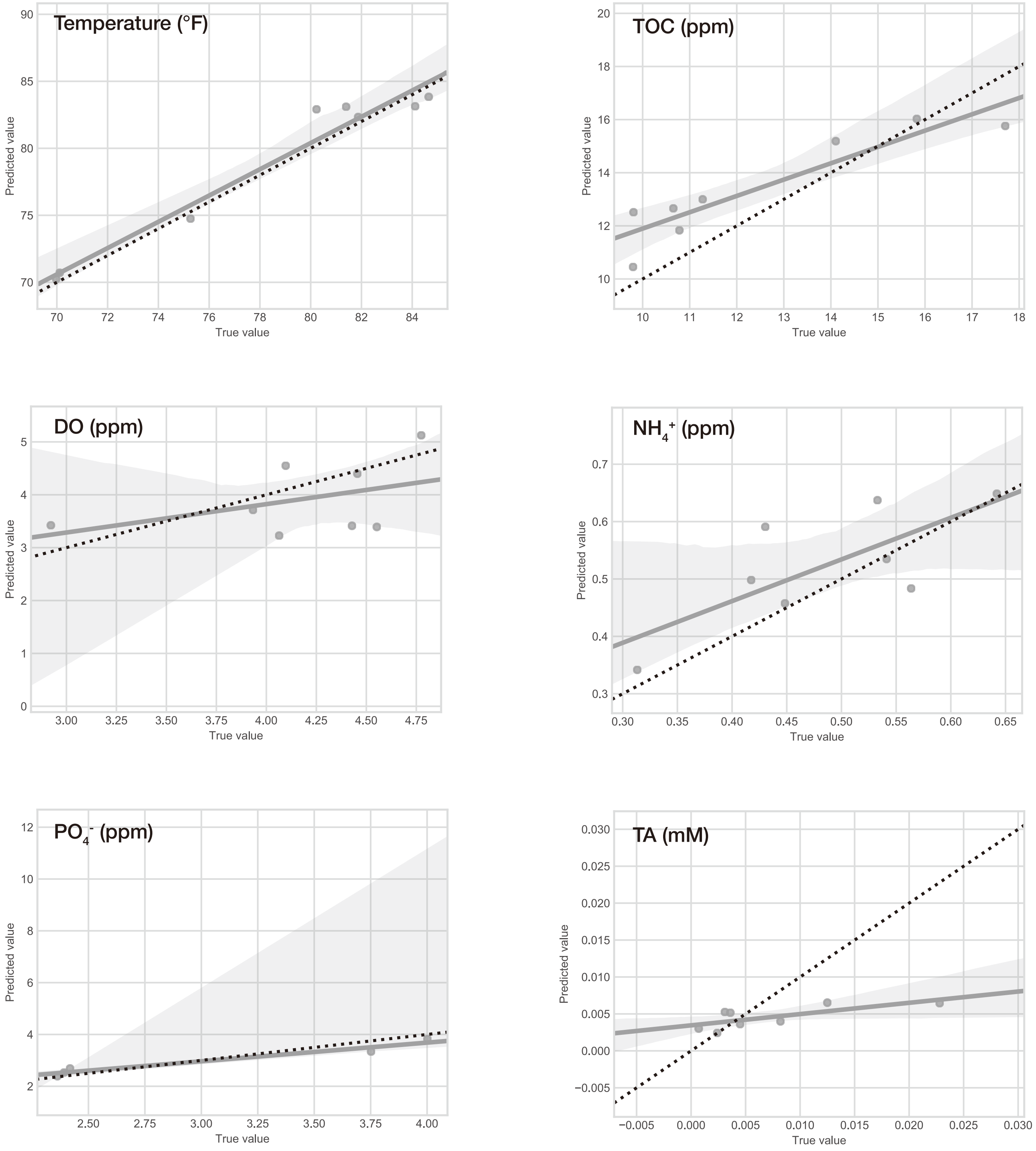

Fig. S8. Prediction of operation parameters based on active community compositions.

Table S1. Selection for fraction of data to train the regressor based on entire community composition

Parameter	Fraction for training	Mean squared error	r-squared	P-value	Std Error	Slope	Intercept
Temperature	0.85	2.023	0.805	0.015	0.190	0.773	18.512
	**0.8**	**9.375**	**0.780**	**0.004**	**0.184**	**0.848**	**12.287**
	0.75	5.871	0.842	0.000	0.136	0.831	14.969
	0.7	6.943	0.846	0.000	0.089	0.625	30.205
	0.65	5.817	0.927	0.000	0.066	0.775	17.373
TOC	0.85	7.819	0.704	0.037	0.143	0.440	7.307
	**0.8**	**7.203**	**0.671**	**0.013**	**0.121**	**0.425**	**7.441**
	0.75	13.677	0.389	0.073	0.135	0.284	10.138
	0.7	21.690	0.621	0.004	0.045	0.174	9.538
	0.65	10.517	0.458	0.011	0.102	0.312	8.429
DO	0.85	1.241	0.193	0.383	0.099	0.096	3.707
	**0.8**	**1.037**	**0.378**	**0.105**	**0.098**	**0.187**	**3.680**
	0.75	0.730	0.146	0.311	0.195	0.213	3.269
	0.7	1.139	0.391	0.040	0.095	0.228	2.939
	0.65	0.519	0.444	0.013	0.113	0.336	2.996
NH4	0.85	0.008	0.387	0.187	0.372	0.591	0.152
	**0.8**	**0.006**	**0.659**	**0.014**	**0.172**	**0.587**	**0.179**
	0.75	0.011	0.224	0.199	0.325	0.462	0.255
	0.7	0.009	0.520	0.012	0.117	0.366	0.301
	0.65	0.015	0.423	0.016	0.132	0.373	0.316
PO4	0.85	0.149	0.999	0.001	0.016	0.662	0.686
	**0.8**	**0.116**	**0.910**	**0.012**	**0.139**	**0.761**	**0.804**
	0.75	0.161	0.840	0.010	0.196	0.897	0.045
	0.7	0.210	0.754	0.005	0.119	0.509	1.281
	0.65	0.674	0.841	0.000	0.033	0.204	1.872
TA	0.85	0.000	0.073	0.603	0.060	0.034	0.004
	**0.8**	**0.000**	**0.120**	**0.400**	**0.138**	**0.125**	**0.002**
	0.75	0.000	0.477	0.039	0.044	0.112	0.003
	0.7	0.000	0.046	0.525	0.078	0.052	0.004
	0.65	0.000	0.297	0.054	0.164	0.353	0.004

Table S2. Proportion of BA+TA in total TOC in the influent to activated sludge

Date	TOC (ppm)	TA (mM)	TA (ppm)	BA (mM)	BA (ppm)	Proportion of BA+TA
16-Feb	304.88	0.42	48.62	1.33	162.11	0.69
11-Apr	457.45	0.39	45.73	1.87	227.61	0.60
9-Jun	486.03	0.28	32.45	1.42	172.68	0.42
13-Jun	469.70	0.39	45.79	1.25	153.04	0.42
14-Jun	427.81	0.33	37.84	1.01	123.62	0.38
19-Jun	491.84	0.30	35.15	0.96	117.47	0.31
